# Chemosynthesis and characterization of site-specific N-terminally PEGylated Alpha-momorcharin as apotential agent

**DOI:** 10.1038/s41598-018-35969-1

**Published:** 2018-12-07

**Authors:** Wenkui Sun, Jinghui Sun, Haowen Zhang, Yanfa Meng, Linli Li, Gangrui Li, Xu Zhang, Yao Meng

**Affiliations:** 1 School of Laboratory Medicine/Sichuan Provincial Engineering Laboratory for Prevention and Control Technology of Veterinary Drug Residue in Animal-origin Food, Chengdu Medical College, Chengdu, 610500 Sichuan China; 2 Department of Chemical and Biological Engineering, University at Buffalo, the State University of New York, Buffalo, New York 14260 United States; 3 Key Laboratory of Bio-resources and Eco-environment Ministry of Education/Animal Disease Prevention and Food Safety Key Laboratory of Sichuan Province, College of Life Science, Sichuan University, Chengdu, 610064 Sichuan China; 4 Department of Pharmaceutics, School of Pharmacy, Chengdu Medical College, Chengdu, 610500 Sichuan China

## Abstract

Alpha-momorcharin (α-MC), a type I ribosome-inactivating protein (RIP) isolated from *Momordica charantia* seeds, has been extensively studied for its antitumor, antiviral and antifungal activities. However, as an exogenous protein, problems associated with short half-life and strong immunogenicity have limited its clinical application. Poly (ethylene glycol) (PEG), as a polyether compound, is a well established and efficient modifier to develop it as a potential agent. Nevertheless, conventional PEGylation is not site-controlled and the conjugates are often not homogenous due to the generation of multi-PEGylated derivatives. To obtain a homogenous mono-PEGylated α-MC, the PEGylation was carried out by coupling a 20 kDa mPEG-butyraldehyde (mPEG-ALD) with α-MC. The product was separated and purified by MacroCap SP chromatography. Results from SDS-PAGE and MALDI-TOF MS revealed that the PEGylated α-MC consisted of one molecule mPEG and α-MC. Edman degradation confirmed that the *N*-terminal residue of α-MC was successfully coupled with mPEG-ALD. The mono-PEGylated α-MC possessed an extremely similar secondary structure to native α-MC through spectral analyses. In addition, it also showed low immunogenicity by double immunodiffusion and preserved moderate antitumor activity to three kinds of tumor cell lines *in vitro*. Finally, trypsin resistance was also considerably improved.

## Introduction

Ribosome-inactivating proteins (RIPs), widely distributed in higher plant tissues, can inactivate eukaryotic ribosomes and therefore specifically and irreversibly inhibit protein synthesis by *N*-glycosidase activity which catalytically cleaves the *N*-glycoside bond at position A^4324^ of the rat liver 28S rRNA^1–7^. As an *N*-glycosidase (EC 3.2.2.22) family of plant toxins, RIPs are mainly divided into three groups^7^. Type I RIPs contains a single polypeptide chain that has a molecular weight of 26–31 kDa and characteristically shows an alkaline pI from 8.0 to 10.0. For example, both trichosanthin (TCS) and pokeweed antiviral protein (PAP) belong to this group of RIPs. Type II presented a dimeric structure consists of two chains which were linked by a disulfide bond. The A chain similar to type I RIPs functions an *N*-glycosidase activity, whereas the B chain has a lectin property which is critical for the binding between protein and target cells^8^. Currently, some researchers have classified RIPs in maize and barley as type III or named typical RIPs, which needs an activation process from inactive precursors (ProRIPs) to active RIPs^9^.

Alpha-momorcharin (α-MC), a single-chain type I RIPs, has been isolated from the seeds of bitter melon *Momordica charantia L*. (MC). It is a glycoprotein and has been confirmed to own several medicinal properties including antitumor, antidiabetic, antimicrobial, antiviral, as well as immune-modulatory both *in vitro* and *in vivo*^10–12^. It consists of about 250 amino acid residues and 1.6% neutral sugar. The relative content of secondary structure is 35.8% of α-helix, 29.9% of β-sheet, 14.7% of β-turn and 19.6% of random coil. The *N*-terminal sequence was N-Asp-Val-Ser-Phe-Arg^13^. More importantly, its strong antitumor and antiviral activity has been attracted a considerable attention which makes it be a potential drug agent^14^. However, like other exogenous proteins, α-MC unfortunately possesses several adverse effects, such as short half-life, strong immunogenicity, toxicity and systemic anaphylaxis^15^. To overcome these problems, various approaches like site mutagenesis and chemical modification have been employed^16,17^. Among them, PEGylation has been found to be one of the effective strategies^18^. Polyethylene glycol (PEG), as a synthetic water-soluble macromolecule, can be covalently attached to therapeutic proteins and owned several benefits^19–21^. In our earlier studies for the non-specific PEGylation with the amino groups on the side chain of lysines and *N*-terminus, the modified α-MC had acceptable bioactivity including longer half-life time in the bloodstream and decreased immunogenicity *in vivo* and *in vitro*^13,22–24^. Although this method is convenient for the formation of the PEGylated conjugates, it often leads to the non-homogenous mixture consisted of other PEGylated byproducts. In order to solve this problem, site-controlled mono-PEGylation of proteins has entered into our view^25^. Currently, many exogenous proteins owned biological activities have been conjugated with various PEG to make them as potential agents for clinical use. For example, Streptokinase (SK) being of bacterial origin owned drawbacks including high antigenicity and relatively short circulating half-life. By using site-specific PEGylation, researchers can obtain longer lasting thrombolytics, which are consistent with clinical requirements^26^. Bovine pancreatic ribonuclease A (RNase) was modified at various extent at the lysine residues by mono-methoxypoly (ethylene glycol) (MPEG). The result showed the half-life was increased of 40–50 folds with respect to the native form^27^. As for ribosome-inactivating proteins, the type 1 RIP Trichosanthin (TCS) has been studied more in these years. It has been approved effective in the clinical treatment of AIDS and tumor, but its strong immunogenicity and short plasma half-life have limited the clinical administration. By using site-directed PEGylation, the PEGylated TCS showed a decrease in immunogenicity, non-specific toxicity, and increase in plasma half-life^17,28,29^. Herein, we report a case study in which chemical synthesis, isolation, identification and *in vitro* bioactivity of mono-PEGylated α-MC were involved. To our knowledge, this was the first study concerning the mono-PEGylation of Alpha-momorcharin as a potential therapeutic agent.

## Results and Discussion

### Preparation of the PEGylated α-MC

**Figure 1:**
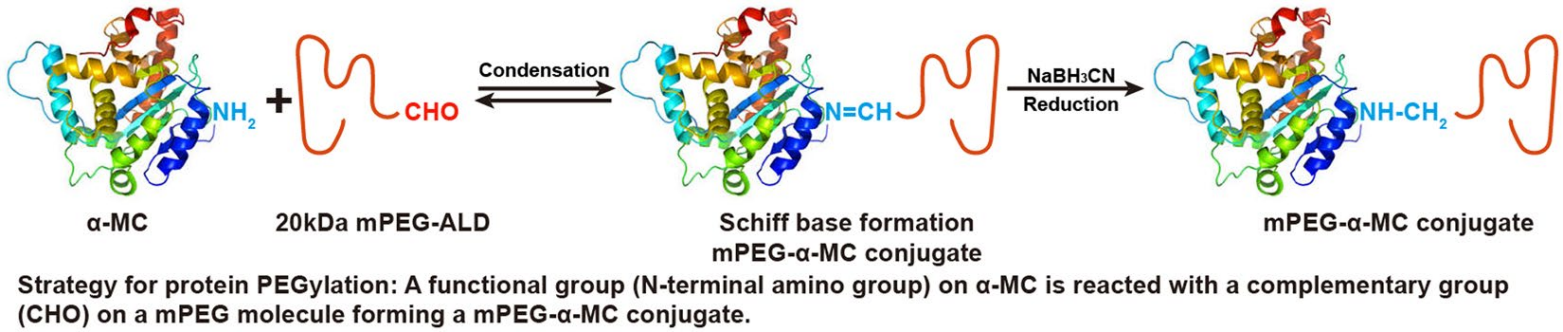
The schematic diagram of chemical synthesis of mono-PEGylated α-MC.

**Figure 2:**
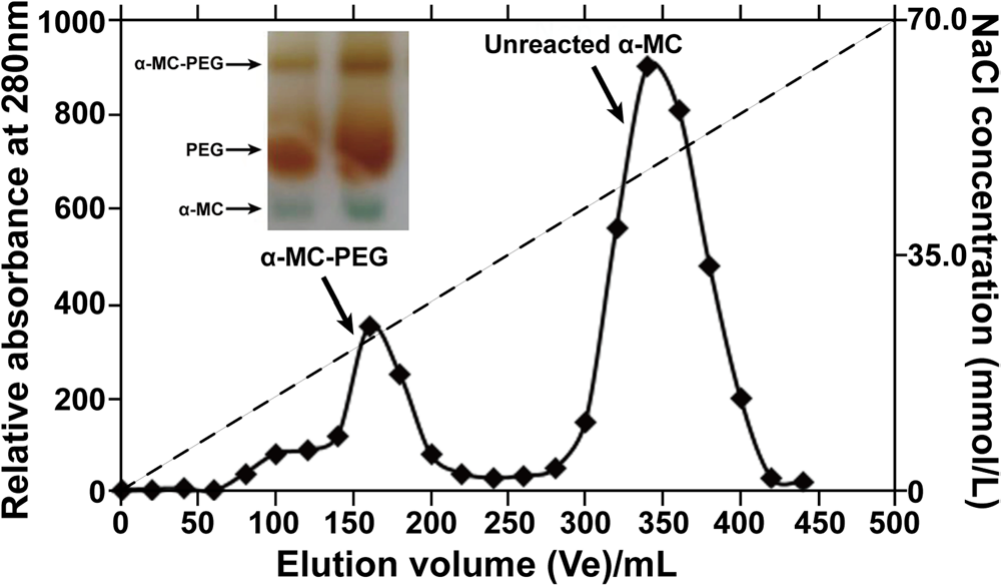
Chromatographic profile of products from α-MC reacted with 20 kDa mPEG-ALD at a mass ratio of PEG: protein as 1.0:3.0 on MacroCAP SP matrix. The column was eluted by 700 mL, pH 6.3, 50 mM NaH_2_PO_4_-Na_2_HPO_4_ buffer with a salt gradient from 0 to 100 mM NaCl. Insert panel indicated the analysis of reaction mixture on SDS-PAGE stained byKI-I solution, from top to bottomwere PEGylated α-MC, residual PEG and unreactive α-MC. The inserted graph was Supplementary Fig. S1.

**Figure 3:**
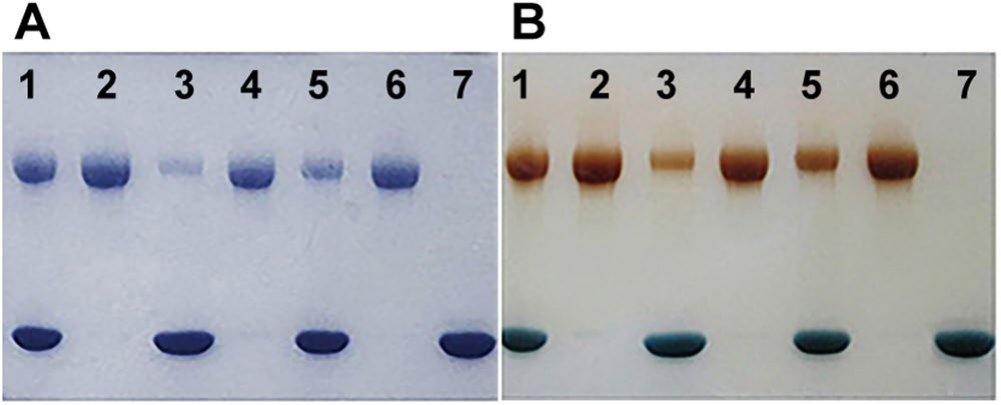
Analytic results of unreacted α-MC and mPEGylated α-MC isolated by MacroCap SP chromatography on SDS-PAGE under reducing or non-reducing conditions. (**A**) represented that the proteinswere stained by coomassie brilliant blue R-250; (**B**) represented that the PEGylated protein and free PEG were stained byKI-I solution. Lane 1,3,5 indicated the PEGylated mixture at a mass ratio of PEG:protein at 1:3.0,1:1.0 and 1:2.0, respectively; Lane 2,4,6 indicated the purified mPEGylated α-MC corresponding to lane 1,3,5; Lane 7 indicated unmodified α-MC.

The PEGylation of proteins depended significantly on the reactive conditions including pH, temperature, the ratio of protein to PEG and reaction time. To obtain mono-PEGylated protein, the conditions should be strictly controlled. In our study, the proposed synthesis scheme (Fig. 1) can obtain an ideal mono-PEGylated protein. The conjugation reactants contained 5.0 mg/mL of α-MC and 20 kDa mPEG-ALD with the mass ratio of PEG-ALD to protein at 1:3, 1:2 and 1:1. The reactants were mixed in pH 4.0, 0.1 M citrate phosphate buffer containing 20 mM sodium cyanoborohydride (NaBH_3_CN) under vibration at 100 r/min at room temperature for 2 h. The reaction was ended by adding 2 M of glycine to a final concentration of 100 mM. The optimal ratio of PEG to protein can be obtained by subsequent SDS-PAGE analysis. A MacroCap SP chromatography was utilized to isolate and purify PEGylated α-MC from the resultant samples containing byproducts which consisted of unmodified protein and unreacted PEG. Once reaction mixture was loaded to the column and residual PEG was removed by elution of pH 6.3, 0.05 M phosphate buffer (buffer A), fractions bounded to media can be eluted by a gradient of 350 mL buffer A: 350 mL buffer A containing 100 mM NaCl. The chromatographic profile was shown in Fig. 2 (Supplementary Fig. S1) and the result of SDS-PAGE in Fig. 3 indicated that the two peaks appeared in order of precedence. The first peak was evidenced to be PEGylated α-MC and the latter one was unreacted α-MC corresponding with a concentration of 20–30 mM and 40–60 mM NaCl, respectively.

A better understanding of the above result may ascribe to the effects of PEGylation on the physicochemical properties of proteins, such as isoelectric point, surface charge density, and distribution, as well as relative hydrophobicity and interactions between PEGylated proteins and surfaces which reduced the interaction between PEGylated protein and media. Additionally, pI of native α-MC and PEGylated one was detected to be 9.04 and 8.68 by IEF-PAGE (data not given here) and this also provided a convincing explanation for the above description.

The homogeneity of PEGylated α-MC was assessed by SDS-PAGE and the result showed a high purity (>95%) without residual PEG appeared at lane 2, 4, 6 in Fig. 3. According to the electrophoresis band distribution of the SDS-PAGE, the purified PEGylated protein possessed about 50% of yield. Meanwhile, it was also shown that the modification rate increased with the increase of the amount of PEG at lane 1, 3, 5. When PEG amount was more than three times of protein, a di-PEGylated form appeared. Therefore, the purified PEGylated protein from a mass ratio of PEG:protein as 1:3 is used in this study.

### MALDI-TOF Mass spectrometry and Edman degradation analysis

**Figure 4:**
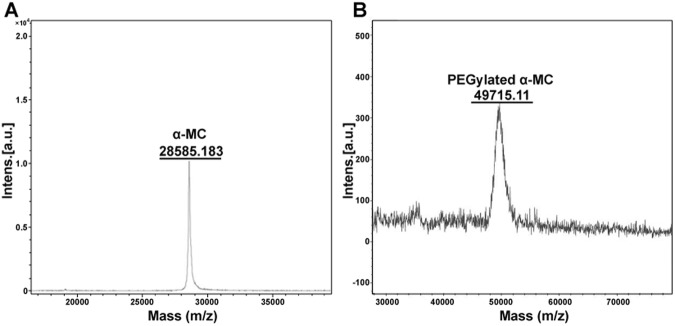
Determination of the accurate molecular weights of native α-MC and the mono-PEGylated α-MC by MALDI-TOF MS. (**A**) presented the analytical result of α-MC and (**B**) presented the analytical result of mono-PEGylated α-MC.

**Figure 5:**
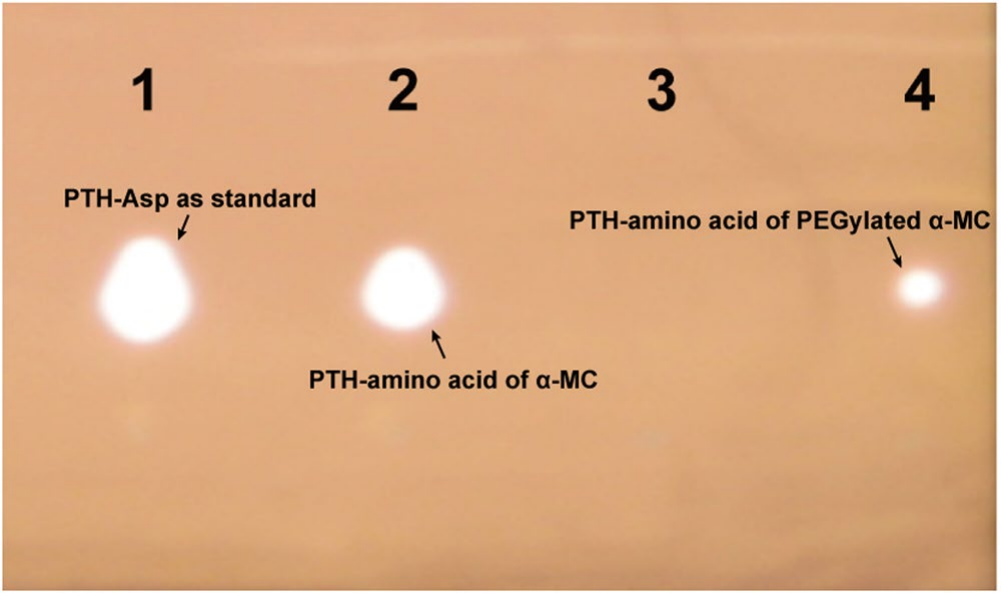
Chromatographic profile of N-terminal amino acid from α-MC and PEGylated α-MC on polyamide film. Lane 1 presented PTH-Asp as standard; Lane 2 presented the PTH-amino acid from α-MC; Lane 3 presented the PTH-amino acid from PEGylated α-MC; Lane 4 presented the PTH-amino acid from PEGylated α-MC with doubling the amount of sample.

In order to verify whether mPEG-ALD was specifically combined with the *N*-terminus of α-MC, a combination of MALDI-TOF MS and Edman Degradation techniques was used. Through the detection of MALDI-TOF MS, the accurate molecular weight of the mPEGylated α-MC was measured to be 49715.11 Da (Fig. 4B) which suggested that a single 20 kDa mPEG-ALD was conjugated to the native α-MC of 28585.183 Da (Fig. 4A). Moreover, chromatographic profile of the PTH-amino acid from α-MC, PTH-amino acid from mPEGylated α-MC and PTH-Asp as standard on polyamide film was shown in Fig. 5. It revealed that the amino acid released from α-MC (Lane 2) was proved to be Asp with the same Rf value as PTH-Asp as standard. But *N*-terminus from PEGylated α-MC could not be released as the PITC would not work if the *N*-terminal amino acid was chemically modified or concealed within the body of the protein (Lane 3). Meanwhile, when the amount of sample was increased to twice, a weak staining spot could be detected in Lane 4 and the reason may due to the possible random modification (positional isomer) to other sites toward α-MC. These data indicated that a 20 kDa mPEG-ALD was indeed conjugated to the *N*-terminus of α-MC by site-specific PEGylation.

### Spectrum analyses

**Figure 6:**
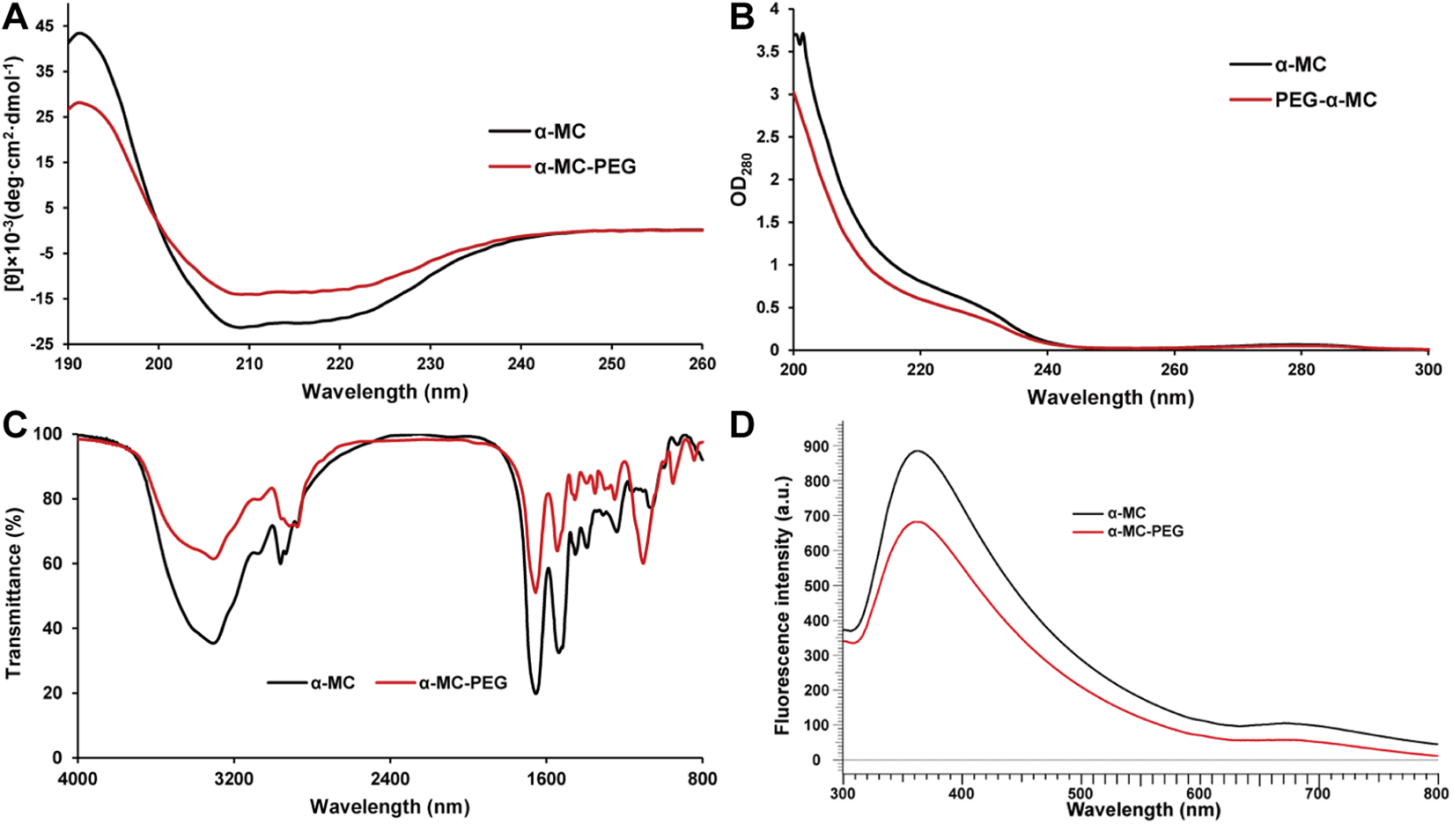
Spectrum analyses of native α-MC and mono-PEGylated α-MC. (**A**) represented circular dichroism (CD); (**B**) represented ultraviolet spectrum (UV); (**C**) represented infrared spectroscopy (IR) and (**D**) represented intrinsic emission fluorescence.

To detect the conformational changes of α-MC in the PEGylation, circular dichroism, UV, IR and FL spectra were analyzed, and the results were presented in Fig. 6. The CD profile (Fig. 6A) of mono-PEGylated α-MC was comparable with that of the native form observed at 205 to 225 nm, indicating helix structure. It was shown that the PEGylation preserved this RIP’s secondary structure. Fluorescence spectra were measured to analyze the differences between the tertiary structure of α-MC and that of mono-PEGylated α-MC. The results were shown in Fig. 6D. The maximum emission wavelength of α-MC was 365 nm, and a blue shift was detected for mono-PEGylated α-MC (360 nm), indicating compact packing of the protein structure and the reason could be attributed to the interaction between PEG and protein. The UV and IR spectra of native α-MC and mono-PEGylated α-MC were approximately similar under detected wavelength (Fig. 6B,C) which showed that the PEGylation does not change the structure of native α-MC.

### Biological activity *in vitro*

Enzymatic activity was carried out by a coupled-enzymes method established in our laboratory. The result indicated that mono-PEGylated protein persisted about 80% activity for catalyzing NAD^+^ as a substrate to release adenine compared to its counterpart.

**Figure 7:**
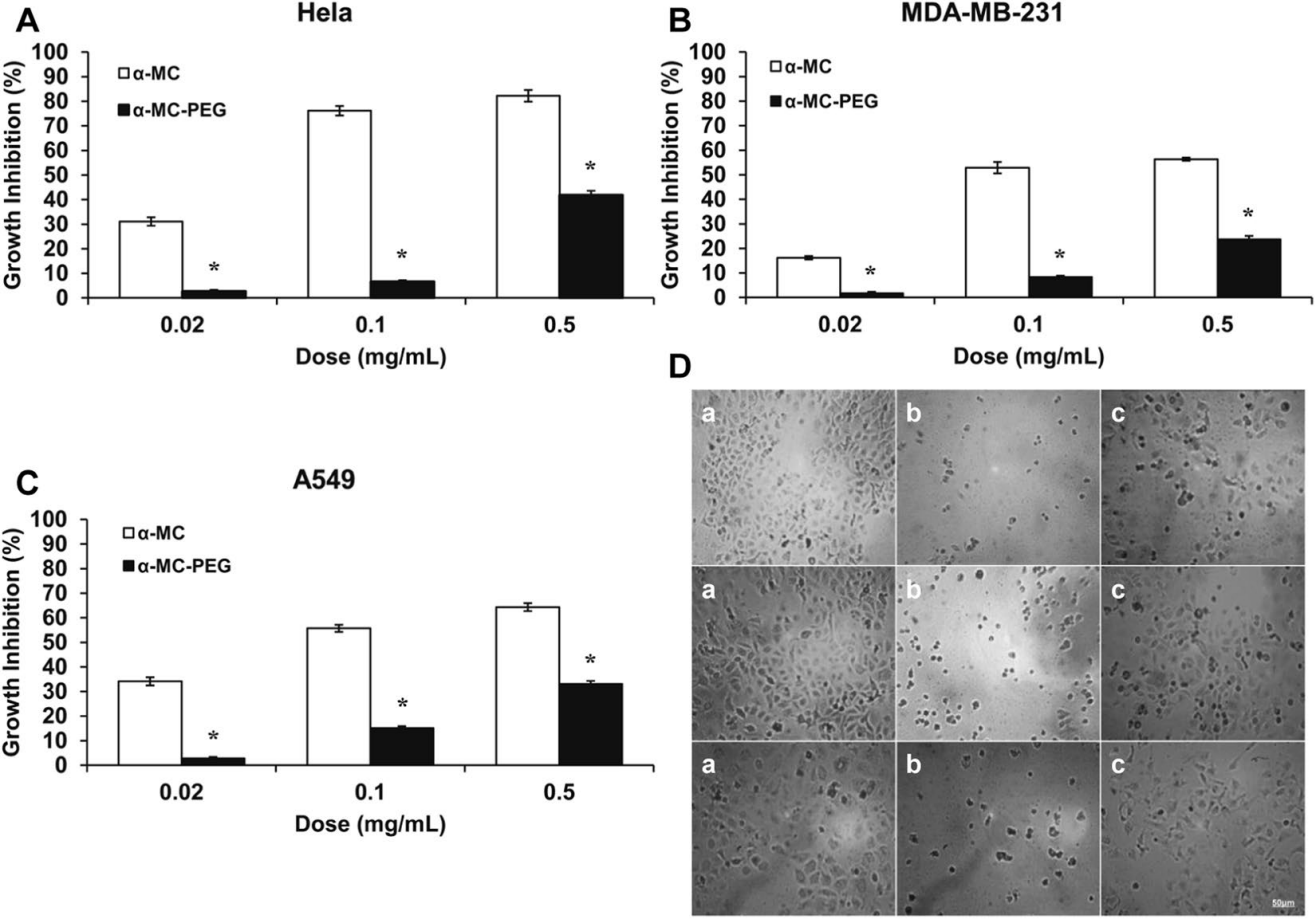
The anti-tumor effects and phase contrast microscope photos of cytotoxicity assay of native α-MC or mono-PEGylated on the proliferation of Hela, MDA-MB-231 and A549 cells for 72 h. (**A**–**C**) Indicated the dose-dependent inhibition of the proliferation of Hela, MDA-MB-231 and A549 cells by native and mono-PEGylated α-MC at different concentrations. Value represented the mean ± SD of three independent experiments. *P < 0.5 compare with α-MC. (**D**) Indicated phase contrast microscope photos of cytotoxicity assay (200x);(a) reflected the normal control of Hela, MDA-MB-231 and A549 cells; (b) reflected Hela, MDA-MB-231 and A549 cells treated with 0.5 mg/mL of α-MC; (c) reflected Hela, MDA-MB-231 and A549 cells treated with 0.5 mg/mL of mono-PEGylated α-MC.

Antitumor effects of native α-MC and mono-PEGylated α-MC were detected by MTT assay. Both exhibited a dose-dependent growth inhibition to Hela, MDA-MB-231 and A549 cell lines at a certain time of 72 h. As Fig. 7 showed, a certain extent of antitumor activity was preserved after PEGylation against these three cell lines with different concentration of native α-MC and mono-PEGylated α-MC. For Hela, MDA-MB-231 and A549 cells, the mono-PEGylate activity was decreased relatively about 91%, 83% and 52% at concentration of 0.02 mg/mL, 0.1 mg/mL and 0.5 mg/mL, respectively. However, cell growth inhibition of them to Hela cells demonstrated a significant decrease, indicating that both α-MC and mono-PEGylated α-MC owned stronger inhibition against human cervix adenocarcinoma cells than against other two cells detected here. Meanwhile, in contrast to the native α-MC, mono-PEGylated α-MC also remained at least 50% of antitumor activity against these three tumor cells at a concentration of 0.5 mg/mL for 72 h. All of these results were similar to our previous studies which used 20 kDa (mPEG)_2_-Lys-NHS or 10 kDa mPEG-SC as the modifiers^22–24,30^. However, the homogeneity of mono-PEGylated-ALD α-MC was much higher than (mPEG)_2_-Lys-NHS α-MC. The reason for this is that we can obtain *N*-terminally PEGylated α-MC according to the difference in pKa values between α-MC group at N-terminus and epsilon amino group of lysine. Generally, the pK of the alpha-amino group is 1–2 pH units lower than the epsilon-amino group of lysine residues. By PEGylating the molecule at pH 7 or below, high selectivity for the N-terminus frequently can be attained^31–33^. It can also be speculated that the structure and molecular weight of PEG have little influence on the antitumor activity of α-MC. Considering other factors, it is also possible that the two types of modifiers did not act on the active center or catalytic group of α-MC, thus the degree of antitumor activity reduction was similar. The result of this part implied us that choosing a PEG molecule with appropriate functional group plays a significant role in PEGylation.

### Analysis of PEGylated α-MC resistance to trypsin proteolysis

**Figure 8:**
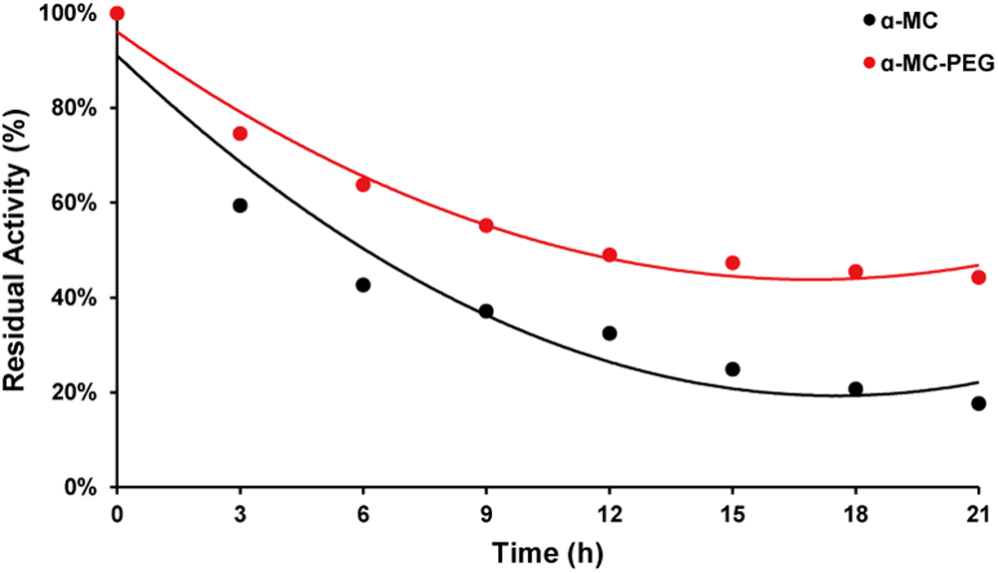
The comparison of resistance to trypsin proteolysis between PEGylated and native α-MC. The proteins were incubated with trypsin for indicated times. Each data value represented means ± SD (n = 3).

α-MC, as an exogenous plant protein, could be degraded in the bloodstream from various serum proteases. Theoretically, the protein stability could be significantly increased by PEGylation. In this section, the comparison of the potent resistance between PEGylated and native α-MC by measuring trypsin resistance *in vitro* was evaluated. The concentration of proteins to trypsin was 2.5:1 and incubated for various time periods. As shown in Fig. 8, the PEGylated α-MC was more resistant to trypsin proteolysis than native α-MC. The activities of them were gradually decreased with the prolongation of the incubation time. After 10 h of treatment with trypsin, nearly 60% of PEGylated α-MC in contrast to 30% of the native was remained, indicating that the PEGylation of α-MC significantly increased its resistance to proteolysis. Additionally, the half-life of PEGylated α-MC was indicated about 16 h while that of α-MC was about 5 h. This may due to the modification-added 20 kDa mPEG-ALD, which can protect the proteolytic sites. Such protection could help this kind of RIPs, α-MC, enter the body and target sites without being hydrolyzed by proteolytic enzymes.

### Double (Ouchterlony) Immunodiffusion

**Figure 9:**
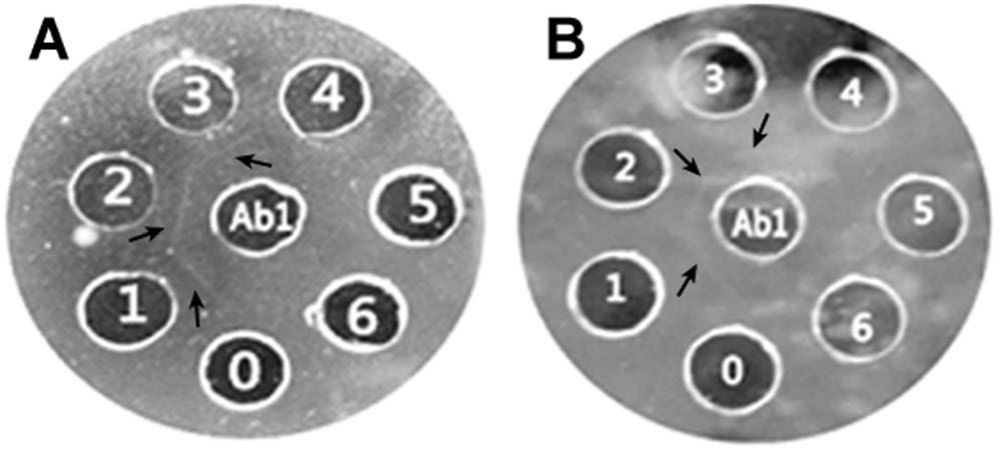
Double immunodiffusion results with α-MC and mono-PEGylated α-MC antisera. (**A**) Result with α-MC antisera at 24 h. Ab1, presented anti-α-MC; 0, presented saline as control; 1–3, presented α-MC (0.5, 0.25, 0.125 mg/mL); 4–6, presented MAP30 (0.5, 0.25, 0.125 mg/mL). (**B**) Result with PEGylated-α-MC antisera at 24 h. Ab1, presented anti-PEGylated-α-MC; 0, presented saline as control; 1–3, presented PEGylated-α-MC (0.5, 0.25, 0.125 mg/mL); 4–6, presented MAP30 (0.5, 0.25, 0.125 mg/mL). Note: arrows pointed the precipitins formed in this test.

The representative of the double immunodiffusion result, Fig. 9 showed Balb/c mice antisera (central well) double immunodiffusion precipitins formed with α-MC, MAP30 (another type 1 RIP purified in our lab) and mono-PEGylated α-MC. Three precipitin lines were observed (Fig. 9A) for α-MC antisera when outer cells were added with 0.5, 0.25 and 0.125 mg/mL of α-MC. Similarly, three precipitin lines were observed (Fig. 9B) for PEGylated α-MC antisera when outer cells were added with 0.5, 0.25 and 0.125 mg/mL of PEGylated α-MC. Notable there is no cross-reactivity between another type 1 RIP named MAP30 with α-MC and PEGylated α-MC antisera. We can conclude that α-MC and MAP30 are different kinds of type 1 RIPs and there is no cross-reactivity. Furthermore, α-MC has a strong precipitin identity with α-MC antisera, while PEGylated α-MC formed with a weaker precipitin. The reason for this may due to the steric hindrance induced by PEG modifier which overlapped the antigen determinant, thus decreasing the immunogenicity.

The attachment of PEG to protein described in this manuscript can be obtained with a single large PEG (mPEG-butyr ALD-20 kDa) at a single site (*N*-terminal amino group). Generally, PEG-conjugated biomolecules exhibited different physicochemical properties including conformational changes, sterical interference, hydrophobicity, and pI. Binding affinities to the receptor binding domains could be often affected by these physicochemical changes, resulting in reduced biological activities in cell-based assays. There is usually a direct relationship between the attached PEG molecular weights and the *in vivo* activity. Meanwhile, an inverse relationship consisted in between the PEG molecules and the *in vitro* activity^34^. To sum up, choosing an appropriate PEG molecule plays a key role in future research.

## Conclusion

In this report, we proposed that mono-PEGylated α-MC can be successfully produced by 20 kDa mPEG-ALD conjugation with α-MC in a weakly acidic pH environment. Although the final product contained some other PEGylated byproducts, we can control the whole purification process and obtain high homogenous mono-PEGylated α-MC by using our purification process described in this manuscript. Through the detections of SDS-PAGE, Edman degradation and MALDI-TOF MS, it was confirmed that *N*-terminus of α-MC was coupled with PEG molecules by covalent manner. The PEGylate can retain original secondary structure by using CD, UV, IR and FL detection, and acceptable antitumor activities against Hela, MDA-MB-231 and A549 cells. Additionally, it also exhibited lower immunogenicity than native α-MC. To sum up, though the site-specific PEGylation of α-MC may offer a possible way for its clinical application as a potential therapeutic agent, we should meet challenges including the reduction of immunogenicity to a great extent, the retention of sufficient biological activity, prolonging the half-life time and development of other types of mono-PEGylated α-MC to make it a useful drug in future. The work described in this manuscript is just the beginning for α-MC to be used clinically. Mono-PEGylation can reduce immunogenicity and retain a certain extent of biological activity, but there still contained lots of problems and lots of work need to be done for further decreasing the immunogenicity and extending the half-life period.

## Methods

### Materials

The mPEG-butyr ALD-20 kDa was purchased from Kaizheng Biotech Development Co. Ltd. (Beijing, China). Fresh bitter melon seeds were obtained from the Institute of Agricultural Science and Technique of Sichuan Province, China. Phenyl isothiocyanate (PITC), adenosine deaminase (ADA), xanthine oxidase (XOD), peroxidase (POD), Aspartic acid(Asp) and sodium cyanoborohydride (NaBH_3_CN) were purchased from Sigma-Aldrich (St Louis, MO). SP-Sepharose FF, Superdex 75, MacroCap^TM^ SP, LMW calibration kit (Phosphorylase b 97.00 kDa; Albumin 66.00 kDa; Ovalbumin 45.00 kDa; Carbonic anhydrase 30.00 kDa; Trypsin inhibitor 20.10 kDa; α-lactabumin 14.4 kDa), ampholyte, pI Calibration Kits (Amyloglucosidase 3.5, Soybean trypsin inhibitor 4.55, β-Lactoglobulin A 5.2, Carbonic anhydrase B bovine 5.85, Carbonic anhydrase B human 6.55, Myoglobin horse 7.35, Lentil lectin-acidic band 8.15, Lentil lectin-middle band 8.45, Lentil lectin-basic band 8.65 and Trypsinogen 9.3) were purchased from GE Healthcare Bio-Sciences AB (Uppsala, SE). Dulbecco’s Modified Eagle’s Medium (DMEM), MTT and fetal bovine serum were from Gibco BRL (Grand Island, NE). The human lung adenocarcinoma A549 cell line (ATCC CCL-185^TM^), the human cervix adenocarcinoma Hela cell line (ATCC CCL-2^TM^) and the human breast adenocarcinoma MDA-MB-231 cell line (ATCC HTB-26^TM^) were obtained from American Type Culture Collection (Manassas, VA). Balb/c mice were purchased from the Laboratory Animal Center of Sichuan University (Chengdu, China). All other chemical reagents were standard commercial products of analytical grade.

### SDS-PAGE and IEF-PAGE

SDS-PAGE was manipulated according to the procedure of Laemmli^35^ on a Mini Protean II apparatus (Bio-Rad, USA). The protein bands were stained by using coomassie brilliant blue R-250 and the PEGylated α-MMC and free PEG were stained with iodine-potassium iodide solution. To estimate the pI of the PEGylated α-MMC, native form and protein markers were determined in thin layer gel slabs with 5.0% polyacrylamide gel and 2.0% ampholyte. Focuses on a Model II Mini Cell was completed under 100 v for 15 min, 200 v for 15 min and 450 v for 60 min. The focused bands were stained by coomassie brilliant blue R-250. The pH gradient curve was obtained by plot of relative migration distance versus pH. According to the relative migration distance of the proteins of interest, the pI value can be found from the pH gradient curve.

### Determination of Protein Concentration

Protein content was according to the method of Lowry *et al*.^36^ or ultraviolent absorbance at 280 nm using human serum albumin (HSA) as standard.

### Isolation and Purification of α-MC

Preparation was carried out according to our previously method with slightly modification^37^. Briefly, all steps in the procedure for preparation of α-MC were carried out at 2–4 °C unless specifically stated. The typical process consists of five steps which yielded homogeneous protein. Firstly, 100 g of fresh and mature bitter melon seeds were decorticated and pulverized with high-speed grinder. The powder was mixed with 500 mL of aceton with −70 °C under stirring and then aceton containing liposoluble substance was removed by centrifugation. The residual aceton in powder was removed by vacuum drying way. The fat-free powder was extracted by adding pH 6.3 0.05 M phosphate buffer (buffer A) containing 0.15 M NaCl under gently stirring overnight. The supernatant was pooled by centrifugation at 15,000 g. Secondly, the supernatant was adjusted to pH 3.6 by adding 0.1 M HCl and then centrifuged. Supernatant was fractionated by 30~60% ammonium sulphate saturation. Thirdly, the crude sample with salt-free by dialysis was loaded to cation-exchange chromatography on SP-Sepharose. Fraction bounded on matrix was collected by elution with buffer A with 0.15 M NaCl. Fourthly, fraction from cation matrix was applied to molecular sieve chromatography on Superdex 75. Finally, fraction with 30 kDa from above step was chromatographed on MacroCap-SP colomn and then α-MC was collected by elution with buffer A containing 55 mM NaCl. The purified α-MC was sterilized through 0.22 µm membrane and stored at 4 °C for subsequent experiments.

### Synthesis of mono-PEGylated α-MC

The synthesis of mono-PEGylation depended significantly on the reactive conditions including pH, temperature, the ratio of protein to PEG and reaction time. According to previous preparation methods^30,38,39^, we used 5.0 mg/mL of α-MC with 20 kDa mPEG-ALD at a mass ratio of PEG-ALD to protein as 1:3, 1:2 and 1:1 in pH 4.0, 0.1 M citrate phosphate buffer containing a catalyst, 20 mM sodium cyanoborohydride (NaBH_3_CN) under vibration at 100 r/min at room temperature for 2 h. The reaction was terminated by adding 2 M of glycine to a final concentration of 100 mM. The modification rate was calculated by SDS-PAGE with Quality One software (Bio-Rad Laboratories) by the following formula:

$${\rm{PEGylation}}\,{\rm{rate}}\,( \% )=(1-\frac{the\,mass\,of\,residual\,\alpha -MC\,in\,the\,reaction\,system}{the\,initial\,mass\,of\,\alpha -MC\,in\,the\,reaction\,system})\times 100 \% $$

The reactive mixture diluted by adding buffer A was applied onto MacroCap SP chromatographic column at a flow rate of 1.0 mL/min and the column was washed by pH 6.3 0.05 M phosphate buffer (buffer A) to eliminate byproducts. The fractions bounded on matrix were eluted by a linear gradient elution from 0 to 100 mM NaCl in buffer A. This part of protein was detected by SDS-PAGE and iodine-potassium iodine analyses^40^.

### Determination of the accurate molecular weight of native α-MC and its mono-PEGylated conjugate

Matrix-Assisted Laser Desorption/Ionization Time-Of-Flight Mass Spectrometry (MALDI-TOF MS) on an Autoflex III (Bruker Corporation, USA) was used^22^. The samples were prepared by mixing with 0.5 μL of aliquot and the matrix solution was 0.5 g/L of α-Cyano-4-hydroxycinnamic acid (α-CCA) in 50% of water/acetonitrile with 0.1% trifluoroacetic acid (TFA). The test was performed by using the positive ion detection mode and the accelerating voltage was 20 000 V as well as a delayed extraction time was 200 ns.

### Identification of *N*-terminal PEGylation

PTH-Asp, PTH-amino acids of α-MC and PEGylated α-MC were prepared according to Fruton *et al*.^41^. Briefly, 0.5 mL(4 mg/mL in H_2_O) Asp reacted with 0.5 mL pyridine and 0.4 mL 10% phenyl isothiocyanate (PITC) at 50 °C for 30 min.7 mg of lyophilized native α-MC and 12 mg of lyophilized mPEGylated α-MC with the same mole ratio were respectively incubated with 0.8 mL of 50% pyridine and 0.2 mL of 10% PITC at 50 °C for 30 min. Samples of further treatments including acid hydrolysis and solvent extraction were stored in ethanol. The same number of samples were loaded onto a polyamide film (7 cm × 7 cm) as a solid phase using PTH-Asp as standard. The chromatography was developed with a mobile phase, n-heptane: pyridine at 7:3 (v/v). When the ascending solvent front neared the top margin, the film was removed and dried. The dried film was soaked in 0.5% starch solution for five minutes and dried again. Finally, PTH- amino acid spots were colored by KI-NaN_3_ as staining reagent.

### Spectrum analyses

The lyophilized α-MC and mPEGylated α-MC were respectively dissolved in ultrapure H_2_O to a concentration of 0.2 mg/mL for immediate use. CD Spectra of them were detected by using a Model Chirascan Plus spectrometer (Applied Photophysics Ltd., UK) in the wavelength range from 190 nm to 260 nm at 20 °C. The quartz cuvette path length was 1 mm and each value were repeated three times. The software package was used for data collection and analysis. In UV analysis, sample solution was scanned by using a Model UV3600 UV/VIS/NIR Spectrophotometer (Hitachi, Japan) in the wavelength range from 200 nm to 300 nm. Each spectrum was repeated three times and the average spectrum was plotted. Intrinsic emission fluorescence spectra of α-MC and mPEGylated α-MC were analyzed by using an F-7000 fluorescence spectrophotometer (Hitachi, Japan). The lyophilized target samples were dissolved in ddH_2_O to 1.0 × 10^−6^ mol/L. Spectra were collected on a 1 cm path-length cuvette with an excitation slit width of 5 nm and an emission slit width of 2.5 nm. The excitation wavelength was set at 280 nm for the specific excitation of tryptophan residues, and emission spectra were recorded from 300 to 800 nm at a constant slit of 1 nm. Each spectrum was done in triplicate and the average spectrum was plotted. For the infrared spectroscopy analysis, about 1 mg of lyophilized α-MC and mPEGylated α-MC was mixed and ground with KBr and then placed under vacuum. The pellets were transferred to IR cells in a nitrogen purged dry box. Target proteins in KBr pellet were scanned from 4000 to 800 nm through Fourier Transform Infrared Spectroscopy (FTIR, Model Nicolet 6700, Thermo Electron Corporation). Each spectrum was scanned three times and the average spectrum was plotted.

### Activity assay for mono-PEGylated α-MC

The depurine activity was measured by a coupling enzyme method which was composed of a combination of adenosine deaminase, xanthine oxidase, peroxidase and cofactor. The adenine quantity that PEGylated α-MC or native α-MC catalyzed NAD^+^ to release was determined. The basic experimental procedure was that 0.5 mL of native α-MC or modified form was reacted with 0.5 mL, 40 mM NAD^+^ in pH 3.0, 0.2 M HAc-NaAc buffer at 55 °C for 30 min. Then the reaction was terminated by treating at 100 °C for 3 min. The reactive mixture was centrifugated at 12000 g for 2 min. 0.5 mL of supernatant was incubated with 2 mL coupled-enzymes color developing solution at 37 °C for 30 min. Absorbance at 555 nm was determined by UV-VIS spectrophotometry. Blank tubes was native α-MC or modified which were heated at 100 °C for 3 min. Other operation process was the same as sample.

Anti-tumor Activity was performed according to Yao *et al*. Three different tumor cell lines which are sensitive to α-MC, including human cervix adenocarcinoma Hela cell line, human lung adenocarcinoma A549 cell line and human breast adenocarcinoma MDA-MB-231 cell line were maintained in DMEM culture medium and supplemented with 10.0% fetal bovine serum containing 100 U/mL penicillin and 50 U/mL streptomycin in a 5.0% CO_2_ incubator at 37.0 °C (Thermo Forma 3110, USA). MTT assay was used to detect the inhibitory effect of α-MC and mPEGylated α-MC. Cell concentration was adjusted to 2.0 × 10^4^ cells/mL and then plated onto the 96-cell plate with a concentration of 100 μL/well. After 12 h initial cultivation, 100 μL of diluted stock solutions of native α-MC and mPEG-α-MC were added at final concentrations of 0.02, 0.1 and 0.5 mg/mL for 72 h (4 replicas per concentration). Cells without adding proteins were used as control. Finally, 20 μL of MTT (5 mg/mL) was added to each cell and placed them to incubate for another 4 h at 37 °C. After adding 100 μL of DMSO and gentle stirring for 10 min, the cells were placed to the plate reader (Model 680, Bio-Rad, USA). The optical density (OD) was measured at a wavelength of 490 nm. The inhibitory effects of α-MC and mPEG- α-MC were analyzed and compared with the control. The cell growth inhibition (%) was calculated with the following formula:

$${\rm{Growth}}\,{\rm{Inhibition}}\,( \% )=\frac{O{D}_{490control}-O{D}_{490sample}}{O{D}_{490control}}\times 100$$

### Proteolytic stability analysis

1.5 mL PEGylated α-MC of 2.5 mg/mL in pH 7.4 0.1 M phosphate buffer was incubated with 1.5 mL trypsin (1.0 mg/mL) at 37 °C and 0.1 mL sample was took out reaction solution at different incubation time. After Each sample diluted appropriately by using pH 3.0, 0.2 M HAc-NaAc buffer, enzymatic activity was determined by a coupling enzyme method described in this paper. Meanwhile the native α-MC was taken as compared with mPEG-α-MC. The profile of residual activity versus reaction time was plotted to investigate the anti-trypsin hydrolytic ability of PEGylated protein.

### Preparation of antiserum and Double (Ouchterlony) Immunodiffusion

The method used here was first described by Ouchterlony in 1948^42^. It is a classic and simple technique that evaluates antibodies in animal or human sera^43,44^. Herein, balb/c mice with both gender (female), 18–21 g, were purchased from the Laboratory Animal Center of Sichuan University (Chengdu China). All mice were housed in a room maintained on an 8:00 a.m. to 8:00 p.m. light cycle at ambient temperature of 23 ± 2 °C with 50–70% humidity. Food and water were provided ad libitum throughout the experiments. Food was withheld 8 h before the experiments. The mice were randomized into 3 groups, with 7 mice in each. 0.25 mg/mL α-MC and mPEGylated α-MC with 0.2 mL in 2 of the 3 groups, 0.2 mL of normal saline in NS control group were subcutaneous administered every 6 days for 4 times. In twenty-third days, mice were killed and the blood was collected and then incubated at 37 °C for 1 h following with centrifugation at 2500 g for 5 min. The prepared antisera were stored at −4 °C. The collected antiserum was added to the central well of the agarose gel plate with 15 μL/well, respectively. 15 μL of diluted antigen, α-MC, mPEGylated α-MC and MAP30 was added to the outer wells, respectively. Plates were incubated at 37 °C for 24 h and the formation of precipitation lines were observed. All the methods of this section were carried out in accordance with the Guideline for Animal Experimentation of Sichuan University (Chengdu, China) and all experimental protocols were approved by the Good Laboratory Practice Regulations for Nonclinical Laboratory Studies issued by Chinese Food and Drug Administration. All the animal care and handling were approved by the Institutional Animal Care and Use Committee of Sichuan University.

### Statistical Analysis

The *in vitro* experiments were performed three times for each individual experiment and all the data were expressed as mean ± SD and subjected to statistical analysis by t-test using SPSS statistical software (SPSS, Inc, Chicago, IL). The difference was considered to be statistically significant when *P* < 0.05.

## Electronic supplementary material

Supplementary Information
