# Supplementary material for: Chemosynthesis and characterization of site-specific N-terminally PEGylated Alpha-momorcharin as apotential agent
Source: Sci Rep. 2018 Dec 7;8:17729. doi: 10.1038/s41598-018-35969-1 (PMC6286350; doi:10.1038/s41598-018-35969-1)

Supplementary Information to

Chemosynthesis and characterization of site-specific N-terminally PEGylated Alpha-momorcharin as a potential agent

Wenkui Sun[1,#], Jinghui Sun[1,#], Haowen Zhang[2], Yanfa Meng[3], Linli Li[3], Gangrui Li[3], Xu Zhang[4], Yao Meng[1, 2*]

[1]School of Laboratory Medicine/Sichuan Provincial Engineering Laboratory for Prevention and Control Technology of Veterinary Drug Residue in Animal-origin Food, Chengdu Medical College, Chengdu 610500, Sichuan, China

[2]Department of Chemical and Biological Engineering, University at Buffalo, the State University of New York, Buffalo, New York 14260, United States

[3]Key Laboratory of Bio-resources and Eco-environment Ministry of Education/Animal Disease Prevention and Food Safety Key Laboratory of Sichuan Province, College of Life Science, Sichuan University, Chengdu 610064, Sichuan, China

[4]Department of Pharmaceutics, School of Pharmacy, Chengdu Medical College, Chengdu 610500, Sichuan, China

[#]These authors contributed equally to this work

[*]Corresponding author:

myaoworks@outlook.com

Figure S1: Full length gel of Figure 2. The analysis of reaction mixture on SDS-PAGE stained by KI-I solution, from top to bottom (Lane 1 & 2) were PEGylated α-MC, residual PEG and unreactive α-MC.

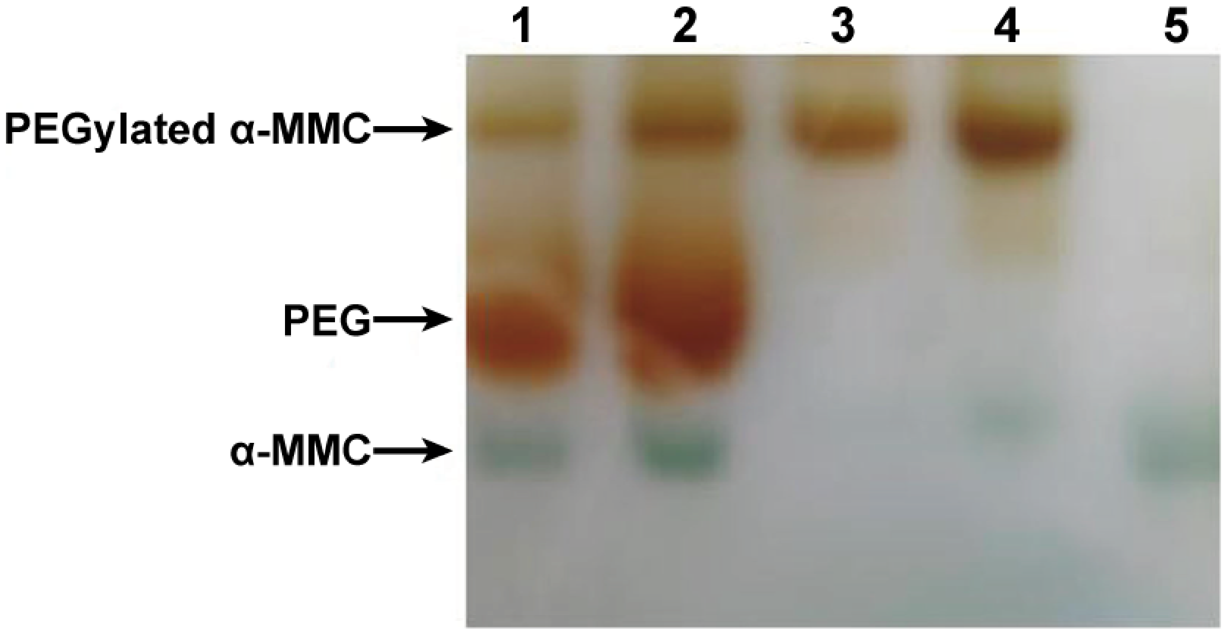

Supplement: Supplementary file 1 — Supplementary Information [file 41598_2018_35969_MOESM1_ESM.pdf]
